# The medication-based Rx-Risk Comorbidity Index and risk of hip fracture - a nationwide NOREPOS cohort study

**DOI:** 10.1186/s12916-024-03335-w

**Published:** 2024-03-13

**Authors:** Siri Marie Solbakken, Haakon Eduard Meyer, Cecilie Dahl, Trine Elisabeth Finnes, Vidar Hjellvik, Christopher Sivert Nielsen, Tone Kristin Omsland, Hein Stigum, Kristin Holvik

**Affiliations:** 1 Department of Physical Health and Ageing, Norwegian Institute of Public Health, Oslo, Norway; 2 Department of Community Medicine and Global Health, Institute of Health and Society, University of Oslo, Oslo, Norway; 3 Section of Endocrinology, Innlandet Hospital Trust, Hamar, Norway; 4 Department of Chronic Diseases, Norwegian Institute of Public Health, Oslo, Norway; 5 Department of Pain Management and Research, Oslo University Hospital, Oslo, Norway

**Keywords:** Hip fracture, Comorbidity index, Multimorbidity, Medication use, Cohort study, Osteoporosis, Epidemiology, Rx-Risk

## Abstract

**Background:**

Few previous studies have assessed overall morbidity at the individual level with respect to future risk of hip fracture. The aim of this register-based cohort study was to examine the association between morbidity measured by the medication-based Rx-Risk Comorbidity Index (Rx-Risk) and the risk of first hip fracture.

**Methods:**

Individual-level data on medications dispensed from pharmacies (2005–2016) was retrieved from the Norwegian Prescription Database and used to calculate Rx-Risk for each calendar year. Information on first hip fractures (2006–2017) was obtained from a nationwide hip fracture database. Individuals ≥ 51 years who filled at least one prescription during the study period comprised the population at risk. Using Rx-Risk as a time-varying exposure variable, relative risk estimates were obtained by a negative binomial model.

**Results:**

During 2006–2017, 94,104 individuals sustained a first hip fracture. A higher Rx-Risk was associated with increased risk of hip fracture within all categories of age and sex. Women with the highest Rx-Risk (> 25) had a relative risk of 6.1 (95% confidence interval (CI): 5.4, 6.8) compared to women with Rx-Risk ≤ 0, whereas the corresponding relative risk in women with Rx-Risk 1–5 was 1.4 (95% CI: 1.3, 1.4). Similar results were found in men. Women > 80 years with Rx-Risk 21–25 had the highest incidence rate (514 (95% CI: 462, 566) per 10, 000 person years). The relative increase in hip fracture risk with higher Rx-Risk was most pronounced in the youngest patients aged 51–65 years.

**Conclusions:**

Rx-Risk is a strong predictor of hip fracture in the general outpatient population and may be useful to identify individuals at risk in a clinical setting and in future studies.

**Supplementary Information:**

The online version contains supplementary material available at 10.1186/s12916-024-03335-w.

## Background

Hip fractures constitute a major healthcare burden, and the absolute numbers are expected to increase in the future due to an aging population [1, 2]. Risk factors for hip fracture have been extensively studied and include various medical conditions [3–8]. A wide range of diagnoses such as rheumatoid arthritis, dementia, diabetes, renal failure, Parkinson’s disease, cardiovascular disease, liver disease, hyperthyroidism, asthma, chronic obstructive pulmonary disease, and cerebrovascular disease are associated with an increased risk of hip fracture [3–6, 8], and morbidity data may improve prediction models for hip fracture risk [4, 9]. However, the number and type of included disease categories vary between studies. Few studies have used a validated morbidity measure to investigate the risk of hip fracture and quantified this risk according to overall morbidity level [8, 10]. As with the Charlson Comorbidity Index, this often requires information on diagnosis codes registered in hospitals, which is not necessarily available in the outpatient population. Alternatively, a medication-based index may be used for this purpose. The Rx-Risk Comorbidity Index (hereafter abbreviated Rx-Risk) is a measure of the morbidity level in an individual based on dispensed prescription medications and can therefore be applied outside the specialist healthcare services. The index has been shown to predict mortality in hip fracture patients [11, 12] and has recently been implemented and validated in the older Norwegian outpatient population [12]. However, we have not been able to identify previous studies using this index to examine hip fracture risk or other disease outcomes. The aims of this study were to investigate the prospective association between the attained medication-based Rx-Risk and the risk of hip fracture and to examine whether this association varied by sex and age.

## Methods

### Study population and design

In this register-based open cohort study, the outcome was defined as an individual’s first hip fracture. Subsequent hip fractures were not included in the analyses. The population at risk included individuals 51 years and older with a Norwegian national identification number who filled at least one prescription at an outpatient pharmacy in Norway during 2005-2016 (*n* = 2,125,367). Participants were followed from the calendar year they turned 51 years old. For each calendar year, Rx-Risk was calculated for all individuals who had filled one or more prescriptions for medication(s) included in the index (see below). Individuals who had not filled prescriptions in an outpatient pharmacy in a calendar year, or who had filled prescriptions only for medications not included in the index, were included in a separate category in the analyses (see sensitivity analysis). As an individual’s Rx-Risk could change during the study period, Rx-Risk was used as a time-varying exposure variable and updated every calendar year. The exposure for each calendar year was the calculated Rx-Risk from the preceding year. Individuals were followed from January 1, 2006, until occurrence of first hip fracture, emigration, death, or end of study on December 31, 2017. Statistics Norway provided information on month and year of death and emigration.

### Hip fractures: the NOREPOS hip fracture database (NORHip)

Information on hospital-treated first hip fractures in Norway sustained in patients aged ≥ 51 years during 2006–2017 was retrieved from the nationwide Norwegian Epidemiologic Osteoporosis Studies (NOREPOS) hip fracture database (NORHip), including month and year of admission. Briefly, hip fractures were defined according to the International Classification of Diseases, Tenth Revision (ICD-10), by the diagnosis codes S72.0-S72.2. Information registered during the hospitalization for hip fracture about primary and secondary diagnosis codes, surgical procedure codes, and time between hospitalizations was used to identify incident hip fractures. For the period 2006–2007 (before the Norwegian Patient Registry could provide identifiable individual data), information on computerized discharge diagnosis codes, surgical procedure codes, and dates of admission were retrieved from the patient administration systems in the hospitals. From 2008 and onwards, the data in NORHip were obtained from the Norwegian Patient Registry. Hip fractures were identified by the same algorithm in both data collection periods, and a thorough validation and quality assurance has been performed. The details regarding data collection, classification of hip fractures, and validation are available [13, 14]. NORHip data from 1994 to 2005 was used as a washout period, increasing the likelihood that only first hip fractures were included. Thus, individuals who sustained a hip fracture during 1994–2005 (before the start of follow-up on January 1, 2006) were excluded.

### Prescription data: the Norwegian Prescription Database (NorPD)

Individual-level data on medications dispensed from outpatient pharmacies in Norway during 2005–2016 (including classification according to the Anatomical Therapeutical Chemical (ATC) Classification System), sex, and birth year were obtained from the Norwegian Prescription Database (NorPD). The NorPD includes medications prescribed in outpatient care with and without reimbursement. Electronic reporting to the NorPD is mandatory, and only drugs collected by the patients are registered. Drugs sold over-the-counter are not included, and medications administered in institutions such as nursing homes and hospitals are not registered on an individual level [15].

### The Rx-Risk Comorbidity Index

The ATC-mapped medication-based Rx-Risk Comorbidity Index has been developed for use in an older outpatient population [16] and has recently been validated in the NorPD with some minor adaptions [12]. In short, it was constructed by assigning medications according to their ATC codes to 46 different disease categories. Each active substance was allocated to one disease category only, based on its main indication, in order to obtain an easily accessible index. Separately for each calendar year, a severity weight for each disease category was assigned based on the odds ratios for mortality in that category, derived from a sex and age-adjusted logistic regression model including indicators of all categories as exposures and death the following year as outcome. Based on the *p*-value and the magnitude of the odds ratios (ORs) for 1-year mortality, each disease category was assigned a severity weight between − 1 and 6 using the following algorithm: weight 0 for any OR with *p*-value > 0.10. For *p*-values ≤ 0.10: weight − 1 for OR < 1, weight 1 for OR 1.00–1.19, weight 2 for OR 1.20–1.39, weight 3 for OR 1.40–1.59, weight 4 for OR 1.60–1.79, weight 5 for OR 1.80–1.99, weight 6 for OR ≥ 2.00 [16]. In our study, the calculation of severity weights was based on calendar-year specific mortality in subjects aged ≥ 50 years who were included in the current study population from the NorPD. Finally, an individual’s total Rx-Risk was calculated separately for each calendar year by summing the weights for all disease categories from which the person had retrieved medication(s) that year. A higher total Rx-Risk indicates greater mortality risk. As a negative severity weight (− 1) was assigned to disease categories with an odds ratio < 1 (indicating a lower relative mortality in patients within those categories), a total Rx-Risk ≤ 0 was possible. The severity weights for each disease category in 2011 (at the approximate mid-point of follow-up) are provided in Additional file 1: Supplementary Table 1. For further details regarding the construction and validation of the index, see Pratt et al. and Holvik et al. [12, 16].

### Statistics

Data were analyzed in Stata SE 17 (Stata Corp., College Station, TX, USA). Graphics were prepared in Microsoft Excel Version 2202 and R version 4.1.0. A calendar year from the middle of the time period (2012) was chosen to represent the descriptive characteristics of the study population. Differences in median Rx-Risk in 2011 (used as exposure for 2012) and median age between hip fracture patients and those without hip fracture in 2012 were tested using a non-parametric equality-of-medians test. Person time was first calculated individually for each calendar year. The number of hip fractures and the person time were then summarized by sex, birth year, calendar year, and Rx-Risk from the preceding year. Incidence rates standardized on birth year and calendar year were calculated by category of Rx-Risk (5-unit intervals, ranging from ≤ 0 to > 25), sex, and attained age (51–65, 66–80, and > 80 years). In model-based analyses of hip fracture risk, a negative binomial model was used as an alternative to Poisson regression due to overdispersion of the data (dispersion parameter alpha 0.03 (95% CI: 0.03, 0.04)) [17]. Sex-stratified incidence rate ratios (IRRs) for hip fracture adjusted for birth year and calendar year were estimated for each Rx-Risk category using the category ≤ 0 as reference. Analyses were further stratified by age groups. Interactions of Rx-Risk with sex, birth year, and calendar year, respectively, were tested. IRRs per 1 unit increase in Rx-Risk were also calculated, using the Rx-Risk as a continuous variable. To investigate sex differences, IRRs for hip fracture in women versus men were estimated by Rx-Risk category and age group. Sex-stratified IRRs for hip fracture were also estimated for the respective disease categories in the Rx-Risk by including each category as an indicator variable (0/1), using disease categories in 2011 as exposure and hip fractures in 2012 as outcome. Within each category, those without the condition (0) were used as reference. Finally, a 1-, 3-, and 5-year latency analysis was performed using Rx-Risk category in 2007 as exposure and first hip fractures in 2008, 2010, and 2012, respectively, as outcomes. In this analysis, individuals with Rx-Risk > 15 were combined in one category due to few cases in single years in those with the highest Rx-Risk.

### Sensitivity analysis

On average, 86% of the patient population filled a prescription for a medication included in the Rx-Risk each calendar year. If an individual had not filled a prescription in a pharmacy for any of the medications included in the index, Rx-Risk could not be calculated for that year. Persons without an Rx-Risk comprised both institutionalized patients as well as younger and presumably healthier individuals who had not received a prescription. For the sake of completeness, individuals without an Rx-Risk were included as a separate category in all analyses. In sensitivity analyses, individuals aged < 70 years without an Rx-Risk were included in the reference category, assuming they constituted a predominantly healthy subgroup of home-dwelling persons who were not currently in need of pharmacological treatment for any chronic condition included in the index. Sensitivity analyses excluding those without an Rx-Risk were also performed.

## Results

### Descriptive characteristics

A total of 2,125,367 individuals were included in the population at risk for one or more calendar years, of whom 94,104 individuals (4.4%) sustained a hip fracture during 2006–2017. Descriptive characteristics of the study population in 2012 are shown in Table 1. Hip fracture patients were older compared with those who did not sustain a hip fracture, and Rx-Risk was higher with a median of 5 (interquartile range (IQR) 2–10) in female hip fracture patients and a median of 2 (IQR 0–6) in women without hip fracture (*p* < 0.001). Similar differences were found in men. Depending on calendar year, individual Rx-Risk values ranged from − 8 to 54. For the majority of the disease categories, the odds ratios for 1-year mortality and the corresponding severity weights were mainly stable during 2005–2016 (Additional file 1: Supplementary Figure 1).

**Table 1:** Descriptive characteristics of individuals ≥ 51 years with and without hip fracture in 2012

	**Hip fracture**	**No hip fracture**
**Women**		
Total number of individuals		
51–65 years	448	439,115
66–80 years	1,532	273,936
> 80 years	3,522	114,936
Total number of person years^a^		
51–65 years	209	438,203
66–80 years	771	271,872
> 80 years	1741	110,034
Median age (IQR)	84 (77–89)	65 (57–74)
Median Rx-Risk in 2011^b^ (IQR)		
51–65 years	4 (0–9)	1 (0–5)
66–80 years	5 (1–10)	2 (0–6)
> 80 years	5 (2–10)	5 (1–8)
**Men**		
Total number of individuals		
51–65 years	356	443,034
66–80 years	813	254,245
> 80 years	1,330	72,602
Total number of person years^a^		
51–65 years	183	441,486
66–80 years	417	251,239
> 80 years	623	68,586
Median age (IQR)	81 (72–87)	63 (57–71)
Median Rx-Risk in 2011^b^ (IQR)		
51–65 years	4 (1–8)	1 (0–5)
66–80 years	5 (1–10)	2 (0–5)
> 80 years	4 (1–9)	3 (0–7)

The Norwegian Prescription Database and the NOREPOS Hip Fracture Database. The calendar year 2012 (at the approximate mid-point of follow-up) was chosen to represent the descriptive characteristics of the study population

^a^Number of person years in 2012

^b^Rx-Risk in 2011 was used as exposure for the calendar year 2012

### Risk of hip fracture by Rx-Risk

Compared to the reference group with Rx-Risk ≤ 0, an increased risk of hip fracture was observed in all Rx-Risk categories and was most pronounced among those with the highest Rx-Risk (Table 2). In analyses adjusted for birth year and calendar year, women with the highest Rx-Risk (> 25) had a more than 6-fold increased risk of hip fracture compared to the reference group (IRR 6.1 (95% CI: 5.4, 6.8)), whereas women with Rx-Risk 1–5 had a 40% increased hip fracture risk (IRR 1.4 (95% CI: 1.3, 1.4)) (Table 2). Similar results were found in men with IRRs varying from 7.1 (95% CI: 6.0, 8.3) for those with an Rx-Risk > 25 to 1.5 (95% CI: 1.4, 1.5) for those with an Rx-Risk of 1–5 (Table 2). Overall, the risk of hip fracture was 6% higher in women and 8% higher in men per unit increase in Rx-Risk (IRR 1.06 (95% CI: 1.06, 1.06) in women and 1.08 (95% CI: 1.08, 1.08) in men, adjusted for birth year and calendar year). No statistical interaction between Rx-Risk and calendar year was observed (*p* = 0.25), but there was a statistical interaction between Rx-Risk and sex (*p* < 0.001) and between Rx-Risk and birth year (*p* < 0.001). In latency analyses, Rx-Risk was still associated with a statistically significant increased hip fracture risk when based on values from 3 and 5 years prior to the calendar year of hip fracture, but the association was somewhat attenuated compared to using the most updated Rx-Risk value (Additional file 1: Supplementary Figure 2).

**Table 2:** Risk of hip fracture by Rx-Risk category in women and men ≥ 51 years

	**Total no. of hip fractures**	**Total no. of person years**	**Incidence rate ratio**^**a**^	**95% CI**
**Women**				
Rx-Risk ≤ 0	10,889	3,372,375	1.0	(ref)
Rx-Risk 1–5	19,141	3,130,998	1.4	(1.3, 1.4)
Rx-Risk 6–10	15,486	1,509,573	1.8	(1.8, 1.9)
Rx-Risk 11–15	7,810	527,277	2.4	(2.3, 2.4)
Rx-Risk 16–20	3,140	158,307	3.1	(3.0, 3.3)
Rx-Risk 21–25	940	38,786	4.1	(3.8, 4.4)
Rx-Risk > 25	289	9,686	6.1	(5.4, 6.8)
Rx-Risk missing^b^	6409	1,050,396	1.6	(1.6, 1.7)
**Men**				
Rx-Risk ≤ 0	5,528	3,024,640	1.0	(ref)
Rx-Risk 1–5	9,515	2,914,461	1.5	(1.4, 1.5)
Rx-Risk 6–10	6,622	1,122,501	2.2	(2.1, 2.3)
Rx-Risk 11–15	3,280	341,269	3.1	(3.0, 3.3)
Rx-Risk 16–20	1,349	96,685	4.3	(4.0, 4.6)
Rx-Risk 21–25	443	25,568	5.4	(4.9, 6.0)
Rx-Risk > 25	142	7,179	7.1	(6.0, 8.3)
Rx-Risk missing^b^	3121	1,514,911	1.7	(1.6, 1.8)

The Norwegian Prescription Database (2005-2016) and the NOREPOS Hip Fracture Database (2006–2017)

^a^Adjusted for birth year and calendar year

^b^Includes individuals who had not filled a prescription for any of the medications included in the Rx-Risk Comorbidity Index in a calendar year

### Absolute and relative risk of hip fracture by Rx-Risk, sex, and age groups

**Fig. 1:**
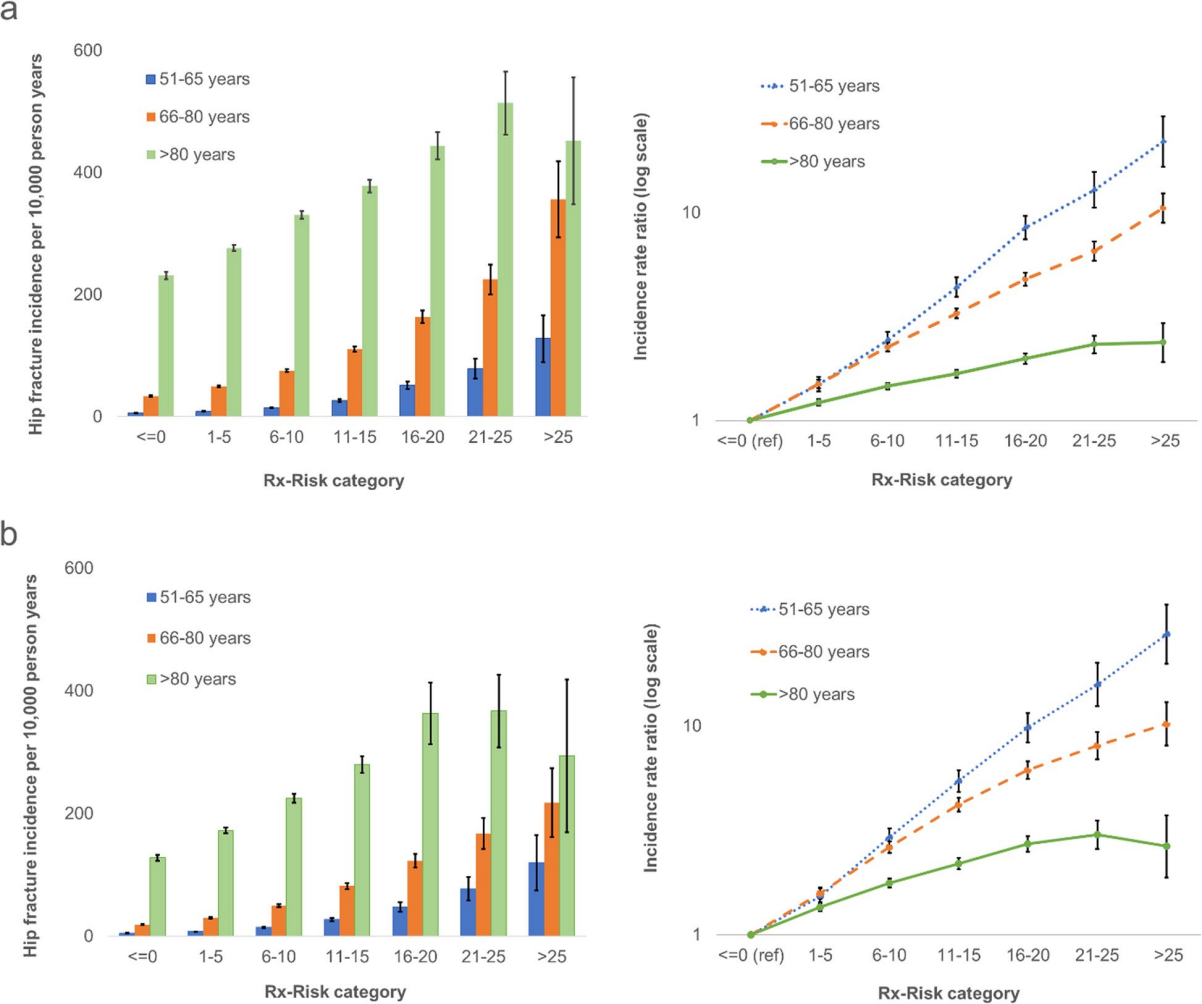
Hip fracture incidence and incidence rate ratios with 95% CIs by Rx-Risk category and age. **a** Women and **b** men. Adjusted for birth year and calendar year. The Norwegian Prescription Database (2005–2016) and the NOREPOS Hip Fracture Database (2006–2017)

A higher Rx-Risk was associated with increasing hip fracture incidence in all age groups for women (Fig. 1a) and men (Fig. 1b). Within each category of Rx-Risk, hip fracture incidence was highest in the oldest (> 80 years) and lowest in the youngest (51–65 years). Hip fracture incidence was also higher in women than in men within all Rx-Risk categories in the age groups 66–80 years and > 80 years. For the age group 51–65 years, incidence rates were similar in men and women within most Rx-Risk categories (Additional file 1: Supplementary Table 2 and Supplementary Figure 3). The highest absolute hip fracture risk was found in women > 80 years with Rx-Risk 21–25 (incidence rate 514 (95% CI: 462, 566) per 10,000 person years). In contrast, the relative increase in hip fracture risk with higher Rx-Risk was most pronounced in the youngest individuals aged 51–65 years (Fig. 1a, b). IRRs among women in this age group varied from 1.5 (95% CI: 1.4, 1.6) for those with Rx-Risk 1–5 to 22.0 (95% CI: 16.7, 29.1 for those with Rx-Risk > 25, when compared to the reference group of the same age with Rx-Risk ≤ 0. The corresponding IRRs in women > 80 years ranged from 1.2 (95% CI: 1.2, 1.3) to 2.4 (95% CI: 1.9, 2.9) (Fig. 1a). Similar patterns were demonstrated in men (Fig. 1b). The risk of hip fracture for each disease category included in the Rx-Risk can be found in Additional file 1: Supplementary Table 3.

### Sensitivity analysis

In sensitivity analyses, persons < 70 years without an Rx-Risk were included in the reference group. This yielded similar results as the main analyses, as did sensitivity analyses excluding those without an Rx-Risk (see Additional file 1: Supplementary Table 4 and 5).

## Discussion

In this population-wide open cohort study from Norway including > 2 million individuals during a 12-year observation period, we found that morbidity level, expressed by the time-varying medication-based Rx-Risk Comorbidity Index, was associated with increased absolute and relative risk of hip fracture within all categories of sex and age. The highest hip fracture incidence rate was found in the oldest women (> 80 years) with a high Rx-Risk. However, the relative increase in hip fracture risk with higher morbidity levels was most pronounced in the youngest patients aged 51–65 years, also reflecting the low absolute hip fracture risk in the reference group of the same age. In addition, it should be noted that at all Rx-Risk levels higher than 0, hip fracture risk was considerably increased compared to the reference group with Rx-Risk ≤ 0.

To our knowledge, this is the first study using the medication-based Rx-Risk Comorbidity Index to investigate the risk of hip fracture. Previous studies have often focused on specific medical conditions and hip fracture risk [3–5], but there are few studies addressing this topic by using a general measure of morbidity level such as a validated index. Two studies have used the Charlson Comorbidity Index based on diagnoses given in specialist healthcare and they both found, in agreement with our results, that a higher comorbidity level was associated with a higher hip fracture risk [8, 10]. In Sweden, a more than twofold increased hip fracture incidence rate among individuals with a Charlson score ≥ 2 compared to a Charlson score of 0 was demonstrated [10]. Furthermore, Reyes et al. found a more than 50% increased risk of hip fracture in men with a Charlson score ≥ 3 compared to men without comorbidities [8]. Neither of these studies reported age-stratified associations between morbidity level and hip fracture risk.

Diagnosis-based versus medication-based indices may vary in their ability to identify medical conditions [18], suggesting that they capture different aspects of morbidity. The diagnosis-based indices may tend to focus on more severe diseases, whereas a medication-based index to a lesser degree distinguishes between disease stages, primary disease prevention, or secondary prophylaxis [19]. However, different treatment indications may represent different patient groups and the risk of hip fracture may vary accordingly between the groups. In addition, more than one condition may be treated with a single drug but will be represented in only one disease category by the Rx-Risk. On the other hand, not all diseases are commonly treated with medications. These will be captured to a lesser extent by the Rx-Risk, for example dementia, which is associated with a higher hip fracture risk [18, 20]. Older, multimorbid patients with short life expectancy may also to a lesser degree receive preventive medication, and some medical conditions could therefore be missed [19]. Consequently, diagnosis-based versus medication-based indices may vary in the degree to which they reflect the underlying medical conditions. The choice of comorbidity index will depend upon the study population and study outcome and naturally the available data. A medication-based index may also complement traditional diagnosis-based indices to provide a more comprehensive assessment of morbidity burden.

When using the Rx-Risk Comorbidity Index, the number and type of prescribed medications is assumed to represent the morbidity level in an individual. However, medication use could also influence hip fracture risk, either by affecting bone mineral density or by increasing fall risk [21–24]. For example, the use of glucocorticoids may induce secondary osteoporosis, and other medications may also be associated with bone loss [21]. Polypharmacy is a risk factor for falling, and some medications such as psychotropic drugs are particularly associated with high fall risk and increased hip fracture risk, even after adjustment for multimorbidity [25–27]. This is especially relevant in an elderly patient population, as older adults are more vulnerable to side effects of medications [28]. Consequently, the pathways for the increased hip fracture risk associated with a high Rx-Risk could partly involve drug side effects, beyond the general morbidity level represented by the index. On the other hand, the different disease categories in Rx-Risk have been weighted according to all-cause mortality risk as each disease category are assigned severity weights between − 1 and 6. An increase in Rx-Risk does not merely reflect an increase in the number of drugs, but rather the severity of the underlying illness(es) in terms of 1-year mortality. As Rx-Risk covers a wide spectrum of medications, beyond drugs affecting bone health or fall risk, it is also less likely that single medications explain the observed associations. We have not been able to identify studies examining the impact of overall morbidity level on hip fracture risk, which have also accounted comprehensively for drug use. However, by design, Rx-Risk is a significant predictor of mortality, reflecting the underlying morbidity represented by the index. A combined effect of morbidity and medications is possible, but it seems less likely that the results in our study can be attributed mainly to medication use. Further studies may be needed to adequately separate the effects of morbidity level and medication side effects on hip fracture risk. Moreover, both the use of medications and hip fracture risk increase with age [29, 30]. In theory, age could have explained the observed association between Rx-Risk and risk of hip fracture if not appropriately accounted for in the analyses. However, in this study, all analyses were adjusted for birth year and calendar year, and in addition analyses by age groups were performed. Thus, Rx-Risk was associated with an increased risk of hip fracture also after accounting for age.

### Benefits of Rx-Risk and further use of the index

Using the Rx-Risk score as a morbidity measure in hip fracture patients has important advantages. It is calculated based on prescription data only and can therefore be used in the home-dwelling population mainly treated in the primary healthcare services, which constitutes a large proportion of the patient population. In contrast, ICD-10 diagnosis-based indices such as the Charlson index may be available only for those who have been in contact with the specialist healthcare services. Moreover, when registering diagnosis codes in hospitals, only diagnoses considered relevant for the current hospitalization are usually registered and some medical conditions could therefore be missed. As the Rx-Risk does not require diagnosis codes, it may be less subject to coding errors compared with diagnosis-based indices. Hence, the Rx-Risk enables the use of a validated morbidity measure in the general outpatient population while taking a comprehensive range of disease categories into account. This medication-based index appears to be a useful indicator of morbidity when studying hip fracture risk in the general population. The current study also suggests the potential for further use of the Rx-Risk when investigating the association between morbidity level and other health outcomes. In the future, Rx-Risk could potentially be calculated and delivered directly from prescription registries to researchers. This would make the Rx-Risk easily accessible and provide a useful tool for comorbidity adjustment in an outpatient study setting. Rx-Risk could also be routinely calculated for elderly patients receiving electronic prescriptions. Thus, the index could help clinicians identify patients at high risk of hip fracture who could benefit from preventive measures.

### Strengths and limitations

The strengths of this registry-based open cohort study include a large sample size based on nationwide registries with mandatory reporting, resulting in near complete information on administrative variables. Due to universal healthcare coverage in Norway, the study population is expected to include the vast majority of the outpatient population. Although drugs sold over-the-counter are not included in the NorPD, there are few medications in Norway that do not require a prescription and these are usually available in small packages only. As treatment guidelines and reimbursement schemes may vary between countries, medication-based indices should ideally be validated when used in a different study setting. The current version of the Rx-Risk has been validated in our study population, and information on hip fractures is based on a thoroughly quality assured national database. Furthermore, Rx-Risk was treated as a time-varying exposure variable and updated every calendar year.

When the Rx-Risk Comorbidity Index was implemented in the patient population from the NorPD, each drug was assigned to one disease category only, in order to obtain a simple and accessible index [12]. However, some drugs have several different indications for treatment representing different disease categories. This will result in some degree of misclassification on an individual level. Additionally, some medications may be given to a patient when needed and not on a regular basis, and this could be more seldom than once per year, depending on the underlying condition and its severity. For some years, this could lead to an underestimation of Rx-Risk for these individuals. As routine medical treatment of chronic conditions typically involves prescriptions dispensed regularly and more frequently than once per year, these exceptions are less likely to have a substantial impact on the estimates. Furthermore, if needed less frequently than once per year, the underlying condition may not represent an important comorbidity.

It is also likely that the frailest old patients are under-represented, as information on medications received in institutions is not included in the NorPD. Due to this limitation, hip fracture incidence rates are probably underestimated in our study, especially in the oldest patient groups with the greatest burden of morbidity. The small subgroup of oldest patients with the very highest Rx-Risk may also represent a special patient group with poor mobility and high 1-year mortality, which could contribute to explain a lower hip fracture incidence in this group. Regarding the patients who did not have an Rx-Risk value for one or more years, we cannot distinguish between institutionalized patients and the presumably healthy group of home-dwelling individuals who had not received a prescription that calendar year. However, the likelihood of belonging to one or the other group will be highly dependent on age. Sensitivity analyses replacing Rx-Risk with 0 in those < 70 years without an Rx-Risk yielded similar results as the main analyses, as did sensitivity analyses excluding those without an Rx-Risk.

In the current study, we have included first hip fractures only with look-back to 1994. It is possible that an individual’s first registered hip fracture could actually represent the second, if the first hip fracture occurred before 1994. However, subsequent hip fractures tend to occur closely in time, often within 5 years [31]. Using the time period 1994–2005 as a washout period was therefore considered acceptable with respect to the risk of misclassifying second hip fractures as first hip fractures.

## Conclusions

In this large, register-based open cohort study, higher morbidity as measured by Rx-Risk was strongly related to increased risk of hip fracture across sex and age groups. The Rx-Risk may be useful to identify patients at high risk of hip fracture in a clinical setting as well as in future studies. As the index does not require diagnosis codes from the specialist healthcare services, it also provides a tool for comorbidity adjustment when investigating hip fracture risk or other health outcomes in the general population. Further studies may be needed to separate the effects of morbidity level and medication use on hip fracture risk.

### Supplementary Information

**Additional file 1:** **Supplementary Table 1-5.** and **Supplementary Figures 1-3.** **Supplementary Table 1.** Disease categories included in the medication-based Rx-Risk Comorbidity Index and their assigned severity weights in 2011, based on individuals aged ≥ 50 years included in the Norwegian Prescription Database. **Supplementary Table 2.** Hip fracture incidence with 95% CIs by Rx-Risk category and age group in women and men. **Supplementary Table 3.** Risk of hip fracture in 2012 in women and men ≥ 51 years by disease categories in 2011 included in the Rx-Risk Comorbidity Index. **Supplementary Table 4.** Sensitivity analyses including individuals <70 years without an Rx-Risk in the reference group. **Supplementary Table 5.** Sensitivity analyses excluding individuals without an Rx-Risk. **Supplementary Figure 1.** Disease categories included in the medication-based Rx-Risk Comorbidity Index and their odds ratios for 1-year mortality and assigned severity weights during 2005-2016, based on individuals aged ≥ 50 years included in the Norwegian Prescription Database. **Supplementary Figure 2.** Latency analysis. Incidence rate ratios with 95% CIs for hip fracture in 2008, 2010 and 2012 by Rx-Risk category in 2007. **Supplementary Figure 3.** Incidence rate ratio of hip fracture in women compared to men by Rx-Risk category and age group.

## Data Availability

The data that support the findings of this study are available upon application to the data owners (the Norwegian Directorate of Health and Statistics Norway). Restrictions apply to the availability of these data, which were used for the purpose of the current study, and so are not publicly available. The corresponding author SMS may be contacted for information about the data and the materials. The code used to perform the analyses is available (DOI: 10.5281/zenodo.10654546)

## Abbreviations

ATC Classification: Anatomical Therapeutical Chemical Classification
CI: Confidence interval
ICD-10: International Classification of Diseases, 10^th^ edition
IQR: Interquartile range
IRR: Incidence rate ratio
NOREPOS: The Norwegian Epidemiologic Osteoporosis Studies
NORHip: The Norwegian Epidemiologic Osteoporosis Studies (NOREPOS) hip fracture database
NorPD: The Norwegian Prescription Database
OR: Odds ratio
Rx-Risk: Rx-Risk Comorbidity Index
