# Supplementary material for: The medication-based Rx-Risk Comorbidity Index and risk of hip fracture - a nationwide NOREPOS cohort study
Source: BMC Med. 2024 Mar 13;22:118. doi: 10.1186/s12916-024-03335-w (PMC10938738; doi:10.1186/s12916-024-03335-w)

The medication-based Rx-Risk Comorbidity Index and risk of hip fracture. A nationwide NOREPOS cohort study

Supplementary Table 1 Disease categories included in the medication-based Rx-Risk Comorbidity Index and their assigned severity weights in 2011, based on individuals aged ≥50 years included in the Norwegian Prescription Database

Disease category	Weight[a]	Disease category	Weight[a]
Alcohol dependency	5	Hyperthyroidism	3
Allergies	-1	Hypothyroidism	-1
Anticoagulants	1	Irritable bowel syndrome	-1
Antiplatelets	0	Ischaemic heart disease: angina	1
Anxiety	3	Ischaemic heart disease: hypertension	0
Arrhythmia	3	Incontinence	-1
Benign prostatic hyperplasia (men only)	-1	Inflammation/pain	-1
Bipolar disorder	1	Liver failure	6
Chronic airways disease	2	Malignancies	6
Congestive heart failure	4	Malnutrition	6
Dementia	4	Migraine	-1
Depression	2	Osteoporosis	-1
Diabetes	2	Pain	5
Epilepsy	4	Pancreatic insufficiency	6
Glaucoma	-1	Parkinson’s disease	2
Gastroesophageal reflux disease	2	Psoriasis	-1
Gout	1	Psychotic illness	4
Hepatitis B	0	Pulmonary hypertension	6
Hepatitis C	0	Renal disease	6
HIV	6	Smoking cessation	2
Hyperkalemia	5	Steroid-responsive disease	6
Hyperlipidemia	-1	Transplant	1
Hypertension	1	Tuberculosis	4

[a] Derived from a logistic regression model with mortality in 2012 as outcome, including age, sex, and indicators (0/1) of each disease category as variables. The severity weights were assigned based on the p-value and the magnitude of the odds ratios (OR) using the following algorithm: Any OR with p-value >0.10: weight 0. For p-values ≤0.10: OR <1: weight -1. OR 1.00 -1.19: weight 1. OR 1.20-1.39: weight 2. OR 1.40-1.59: weight 3. OR 1.60-1.79: weight 4. OR 1.80-1.99: weight 5. OR ≥2.00: weight 6

Supplementary Table 2 Hip fracture incidence with 95% CIs by Rx-Risk category and age group in women and men. Adjusted for birth year and calendar year. The Norwegian Prescription Database (2005-2016) and the NOREPOS Hip Fracture Database (2006-2017)

**Women**	**Total number of hip fractures**	**Hip fracture incidence per 10 000 person years**	**95% CI**
**51-65 years**			
Rx-Risk Score ≤0	1184	6	(6, 6)
Rx-Risk Score 1-5	1429	9	(8, 9)
Rx-Risk Score 6-10	967	15	(14, 15)
Rx-Risk Score 11-15	530	26	(24, 29)
Rx-Risk Score 16-20	293	51	(45, 58)
Rx-Risk Score 21-25	110	79	(63, 95)
Rx-Risk Score >25	52	128	(89, 166)
**66-80 years**			
Rx-Risk Score ≤0	3431	34	(33, 35)
Rx-Risk Score 1-5	5552	50	(49, 51)
Rx-Risk Score 6-10	4407	75	(73, 78)
Rx-Risk Score 11-15	2413	111	(106, 115)
Rx-Risk Score 16-20	1097	164	(153, 174)
Rx-Risk Score 21-25	377	225	(201, 250)
Rx-Risk Score >25	151	356	(293, 418)
**>80 years**			
Rx-Risk Score ≤0	6274	231	(225, 237)
Rx-Risk Score 1-5	12160	277	(272, 282)
Rx-Risk Score 6-10	10112	331	(324, 337)
Rx-Risk Score 11-15	4867	378	(367, 389)
Rx-Risk Score 16-20	1750	444	(422, 466)
Rx-Risk Score 21-25	453	514	(462, 566)
Rx-Risk Score >25	86	452	(348, 556)

Supplementary table 2 cont.

Men	Total number of hip fractures	Hip fracture incidence per 10 000 person years	95% CI
**51-65 years**			
Rx-Risk Score ≤0	884	5	(5, 5)
Rx-Risk Score 1-5	1193	8	(7, 8)
Rx-Risk Score 6-10	789	14	(13, 15)
Rx-Risk Score 11-15	392	27	(24, 30)
Rx-Risk Score 16-20	182	48	(40, 55)
Rx-Risk Score 21-25	74	77	(58, 96)
Rx-Risk Score >25	38	120	(75, 165)
**66-80 years**			
Rx-Risk Score ≤0	1910	19	(18, 20)
Rx-Risk Score 1-5	3177	30	(29, 31)
Rx-Risk Score 6-10	2233	50	(48, 52)
Rx-Risk Score 11-15	1204	82	(77, 87)
Rx-Risk Score 16-20	539	123	(112, 134)
Rx-Risk Score 21-25	196	168	(142, 193)
Rx-Risk Score >25	71	218	(162, 274)
**>80 years**			
Rx-Risk Score ≤0	2734	128	(123, 133)
Rx-Risk Score 1-5	5145	173	(168, 177)
Rx-Risk Score 6-10	3600	225	(218, 232)
Rx-Risk Score 11-15	1684	280	(266, 294)
Rx-Risk Score 16-20	628	363	(313, 413)
Rx-Risk Score 21-25	173	367	(308, 426)
Rx-Risk Score >25	33	294	(170, 419)

Supplementary Table 3 Risk of hip fracture in 2012 in women and men ≥51 years by disease categories in 2011 included in the Rx-Risk Comorbidity Index. Disease categories are ordered from the highest to the lowest incidence rate ratio. The Norwegian Prescription Database and the NOREPOS Hip Fracture Database.

**WOMEN**			
**Disease category**	**Severity weight[a]**	**Incidence rate ratio[b]**	**95% CI**
Dementia	4	2.55	(2.21, 2.94)
Parkinson’s disease	2	1.82	(1.49. 2.22)
Malignancies	6	1.76	(1.16, 2.66)
Liver failure	6	1.76	(1.38, 2.23)
Psychotic illness	4	1.71	(1.52, 1.92)
Renal disease	6	1.67	(1.15, 2.43)
Transplant	1	1.65	(0.78, 3.49)
Pancreatic insufficiency	6	1.46	(0.95, 2.24)
Alcohol dependency	5	1.45	(0.45, 4.75)
Epilepsy	4	1.45	(1.27, 1.66)
Depression	2	1.42	(1.31, 1.54)
Malnutrition	6	1.40	(0.17, 11.53)
Tuberculosis	4	1.40	(0.30, 6.51)
Irritable bowel syndrome	-1	1.34	(0.97, 1.84)
Chronic airways disease	2	1.30	(1.19, 1.42)
Diabetes	2	1.27	(1.14, 1.41)
Anticoagulants	1	1.27	(1.14, 1.40)
Pain	5	1.19	(1.11, 1.28)
Anxiety	3	1.17	(1.08, 1.26)
Hyperthyroidism	3	1.16	(0.72, 1.86)
Antiplatelets	0	1.15	(1.07, 1.23)
Steroid-responsive disease	6	1.14	(1.04, 1.26)
Congestive heart failure	4	1.14	(1.00, 1.31)
Psoriasis	-1	1.12	(0.83, 1.53)
Gastrooesophageal reflux disease	2	1.07	(0.99, 1.16)
Incontinence	-1	1.02	(0.89, 1.16)
Glaucoma	-1	1.01	(0.91, 1.11)
Ischaemic heart disease: hypertension	0	1.00	(0.93, 1.07)
Smoking cessation	2	0.98	(0.64, 1.50)
Allergies	-1	0.97	(0.89, 1.07)
Arrhythmia	3	0.96	(0.83, 1.12)
Ischaemic heart disease: angina	1	0.95	(0.85, 1.06)
Osteoporosis	-1	0.93	(0.84, 1.03)
Hypertension	1	0.89	(0.83, 0.95)
Hypothyroidism	-1	0.86	(0.79, 0.94)
Hyperlipidaemia	-1	0.83	(0.77, 0.90)
Gout	1	0.79	(0.65, 0.97)

WOMEN			
**Disease category**	**Severity weight[a]**	**Incidence rate ratio[b]**	**95% CI**
Inflammation/pain	-1	0.79	(0.73, 0.85)
Medications not in the index[c]		0.77	(0.64, 0.93)
Migraine	-1	0.76	(0.56, 1.02)
Bipolar disorder	1	0.74	(0.41, 1.32)
HIV[d]	6	-	
Hepatitis C[d]	0	-	
Hepatitis B[d]	0	-	
Hyperkalaemia[d]	5	-	
Pulmonary hypertension[d]	6	-	

MEN			
**Disease category**	**Severity weight[a]**	**Incidence rate ratio[b]**	**95% CI**
HIV	6	4.69	(1.38, 15.93)
Dementia	4	3.13	(2.47, 3.97)
Malnutrition	6	2.80	(0.23, 33.36)
Parkinson's disease	2	2.71	(2.12, 3.48)
Alcohol dependency	5	2.33	(1.13, 4.80)
Pancreatic insufficiency	6	2.10	(1.13, 3.92)
Renal disease	6	2.05	(1.36, 3.09)
Transplant	1	1.93	(0.99, 3.77)
Depression	2	1.78	(1.55, 2.05)
Epilepsy	4	1.74	(1.44, 2.10)
Malignancies	6	1.72	(1.00, 2.98)
Psychotic illness	4	1.68	(1.37, 2.06)
Pain	5	1.59	(1.43, 1.77)
Hyperthyroidism	3	1.57	(0.44, 5.59)
Smoking cessation	2	1.50	(0.92, 2.43)
Liver failure	6	1.40	(0.97, 2.02)
Chronic airways disease	2	1.33	(1.17, 1.51)
Irritable bowel syndrome	-1	1.31	(0.82, 2.09)
Bipolar disorder	1	1.26	(0.62, 2.56)
Anticoagulants	1	1.26	(1.09, 1.44)
Osteoporosis	-1	1.25	(0.90, 1.75)
Antiplatelets	0	1.24	(1.11, 1.39)
Anxiety	3	1.23	(1.07, 1.43)
Steroid-responsive disease	6	1.22	(1.04, 1.41)
Congestive heart failure	4	1.17	(0.96, 1.41)
Glaucoma	-1	1.06	(0.90, 1.24)
Gastrooesophageal reflux disease	2	1.05	(0.93, 1.18)
Incontinence	-1	1.03	(0.80, 1.32)
Diabetes	2	1.00	(0.86, 1.16)
Hypertension	1	0.96	(0.87, 1.07)
Ischaemic heart disease: hypertension	0	0.96	(0.87, 1.07)

MEN			
**Disease category**	**Severity weight[a]**	**Incidence rate ratio[b]**	**95% CI**
Gout	1	0.92	(0.75, 1.13)
Hypothyroidism	-1	0.91	(0.73, 1.12)
Benign prostatic hyperplasia (men only)	-1	0.91	(0.79, 1.04)
Psoriasis	-1	0.88	(0.57, 1.36)
Arrhythmia	3	0.88	(0.71, 1.08)
Ischaemic heart disease: angina	1	0.87	(0.73, 1.02)
Medications not in the index[c]		0.85	(0.66, 1.10)
Allergies	-1	0.80	(0.68, 0.93)
Hyperkalaemia	5	0.70	(0.14, 3.46)
Inflammation/pain	-1	0.69	(0.61, 0.79)
Hyperlipidaemia	-1	0.69	(0.61, 0.77)
Migraine	-1	0.24	(0.08, 0.78)
Tuberculosis[d]	4	-	
Hepatitis C[d]	0	-	
Hepatitis B[d]	0	-	
Pulmonary hypertension[d]	6	-	

[a] Severity weight in 2011

[b] Each disease category was included as an indicator variable (0/1), using disease categories in 2011 as exposure and hip fractures in 2012 as outcome. Within each category, those without the condition (0) were used as reference. Adjusted for birthyear and the other categories in the table

[c] Includes medications such as hypnotics/sedatives, oestrogens, antibiotics/anti-infectives, mineral supplements, cough and cold preparations. This category is not part of the Rx-Risk and has no severity weight, but the individuals are registered in the Norwegian Prescription Database and are part of the background population count.

[d] Disease categories with no hip fractures in 2012

Supplementary Table 4 Sensitivity analysis including individuals <70 years without an Rx-Risk in the reference group. Risk of hip fracture by Rx-Risk category in women and men ≥51 years, based on the Norwegian Prescription Database (2005-2016) and the NOREPOS Hip Fracture Database (2006-2017)

		Incidence rate ratio [a]	95% CI
**Women**			
	Rx-Risk ≤0[b]	1.0	(ref)
	Rx-Risk 1-5	1.4	(1.3, 1.4)
	Rx-Risk 6-10	1.8	(1.8, 1.9)
	Rx-Risk 11-15	2.4	(2.3, 2.4)
	Rx-Risk 16-20	3.1	(3.0, 3.3)
	Rx-Risk 21-25	4.1	(3.8, 4.4)
	Rx-Risk >25	6.1	(5.4, 6.8)
	Rx-Risk missing[c]	1.9	(1.8, 1.9)
**Men**			
	Rx-Risk ≤0[b]	1.0	(ref)
	Rx-Risk 1-5	1.5	(1.4, 1.5)
	Rx-Risk 6-10	2.2	(2.1, 2.3)
	Rx-Risk 11-15	3.1	(2.9, 3.2)
	Rx-Risk 16-20	4.3	(4.0, 4.5)
	Rx-Risk 21-25	5.4	(4.9, 5.9)
	Rx-Risk >25	7.0	(5.9, 8.2)
	Rx-Risk missing[c]	2.1	(1.9, 2.2)

[a] Adjusted for birth year and calendar year
[b] For the purpose of sensitivity analysis, individuals <70 years without an Rx-Risk were included in the reference group
[c] Includes individuals ≥70 years who had not filled a prescription for any of the medications included in the Rx-Risk Comorbidity Index in a calendar year

Supplementary Table 5 Sensitivity analysis excluding individuals without an Rx-Risk. Risk of hip fracture by Rx-Risk category in women and men ≥51 years, based on the Norwegian Prescription Database (2005-2016) and the NOREPOS Hip Fracture Database (2006-2017)

		Incidence rate ratio [a]	95% CI
**Women**			
	Rx-Risk ≤0	1.0	(ref)
	Rx-Risk 1-5	1.4	(1.3, 1.4)
	Rx-Risk 6-10	1.8	(1.8, 1.9)
	Rx-Risk 11-15	2.3	(2.3, 2.4)
	Rx-Risk 16-20	3.1	(3.0, 3.2)
	Rx-Risk 21-25	4.1	(3.8, 4.3)
	Rx-Risk >25	6.0	(5.4, 6.8)
**Men**			
	Rx-Risk ≤0	1.0	(ref)
	Rx-Risk 1-5	1.5	(1.4, 1.5)
	Rx-Risk 6-10	2.2	(2.1, 2.3)
	Rx-Risk 11-15	3.2	(3.0, 3.3)
	Rx-Risk 16-20	4.3	(4.1, 4.6)
	Rx-Risk 21-25	5.5	(5.0, 6.1)
	Rx-Risk >25	7.1	(6.0, 8.4)

[a]Adjusted for birth year and calendar year

Supplementary Figure 1 Disease categories included in the medication-based Rx-Risk Comorbidity Index and their odds ratios for 1-year mortality (dotted red lines) and assigned severity weights[a] (dotted black lines) during 2005-2016, based on individuals aged ≥50 years included in the Norwegian Prescription Database

[a] Derived from a logistic regression model with 1-year mortality as outcome, including age, sex, and indicators (0/1) of each disease category as variables. The severity weights were assigned based on the p-value and the magnitude of the odds ratios (OR) using the following algorithm: Any OR with p-value >0.10: weight 0. For p-values ≤0.10: OR <1: weight -1. OR 1.00 -1.19: weight 1. OR 1.20-1.39: weight 2. OR 1.40-1.59: weight 3. OR 1.60-1.79: weight 4. OR 1.80-1.99: weight 5. OR ≥2.00: weight 6

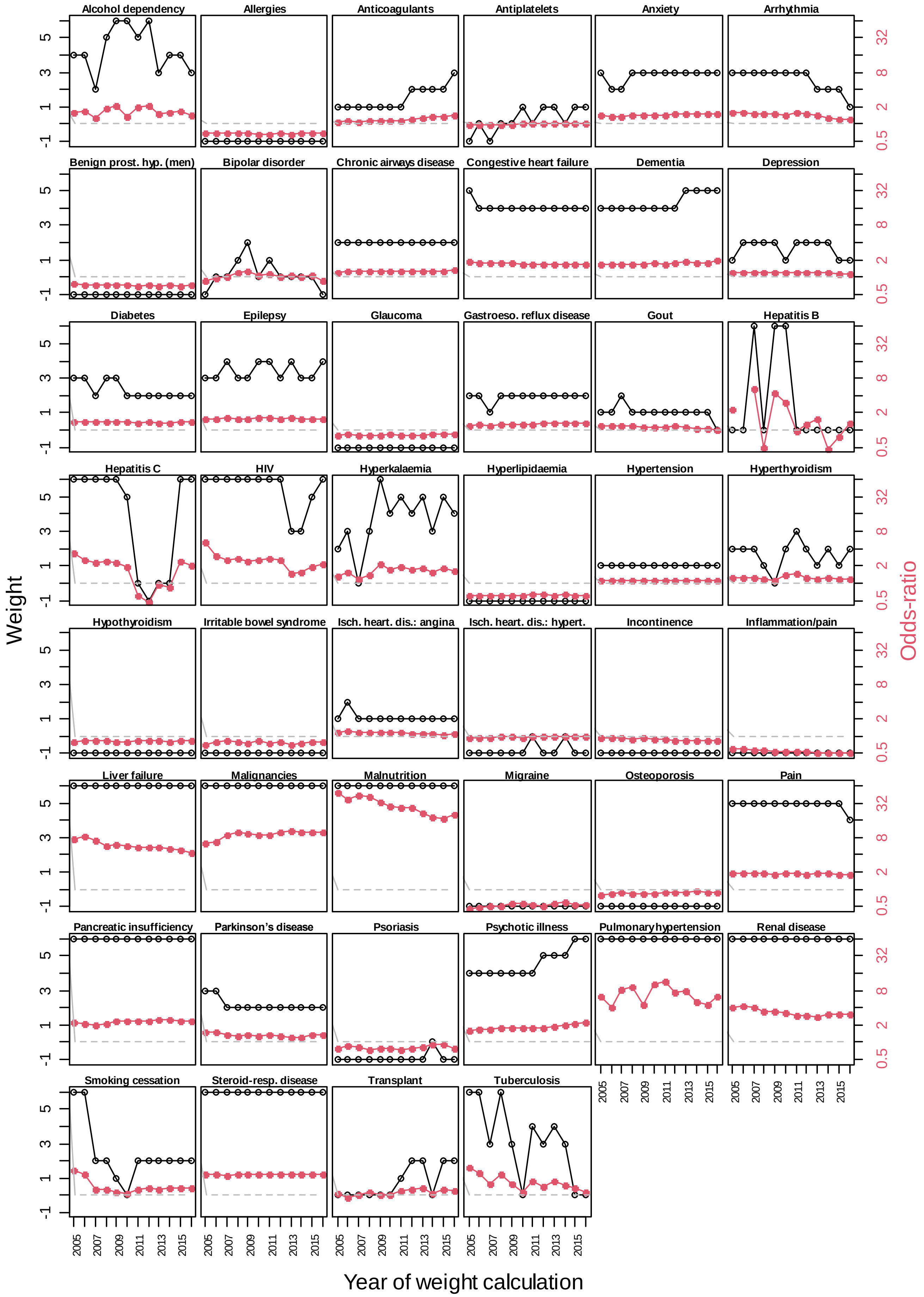
Alcohol dependency
Allergies
Anticoagulants
Antiplatelets
Anxiety
Arrhythmia
Benign prost. hyp. (men)
Bipolar disorder
Chronic airways disease
Congestive heart failure
Dementia
Depression
Diabetes
Epilepsy
Glaucoma
Gastroeso. reflux disease
Gout
Hepatitis B
Hepatitis C
HIV
Hyperkalaemia
Hyperlipidaemia
Hypertension
Hyperthyroidism
Hypothyroidism
Irritable bowel syndrome
Isch. heart. dis.: angina
Isch. heart. dis.: hypert.
Incontinence
Inflammation/pain
Liver failure
Malignancies
Malnutrition
Migraine
Osteoporosis
Pain
Pancreatic insufficiency
Parkinson's disease
Psoriasis
Psychotic illness
Pulmonary hypertension
Renal disease
Smoking cessation
Steroid-resp. disease
Transplant
Tuberculosis
Weight
Odds-ratio
Year of weight calculation

Supplementary Figure 2 Latency analysis. Incidence rate ratios (IRRs) with 95% CIs for hip fracture in 2008, 2010 and 2012 by Rx-Risk category in 2007 in a) women and b) men. Rx-Risk ≤0 was used as reference. Adjusted for birthyear. The Norwegian Prescription Database and the NOREPOS Hip Fracture Database

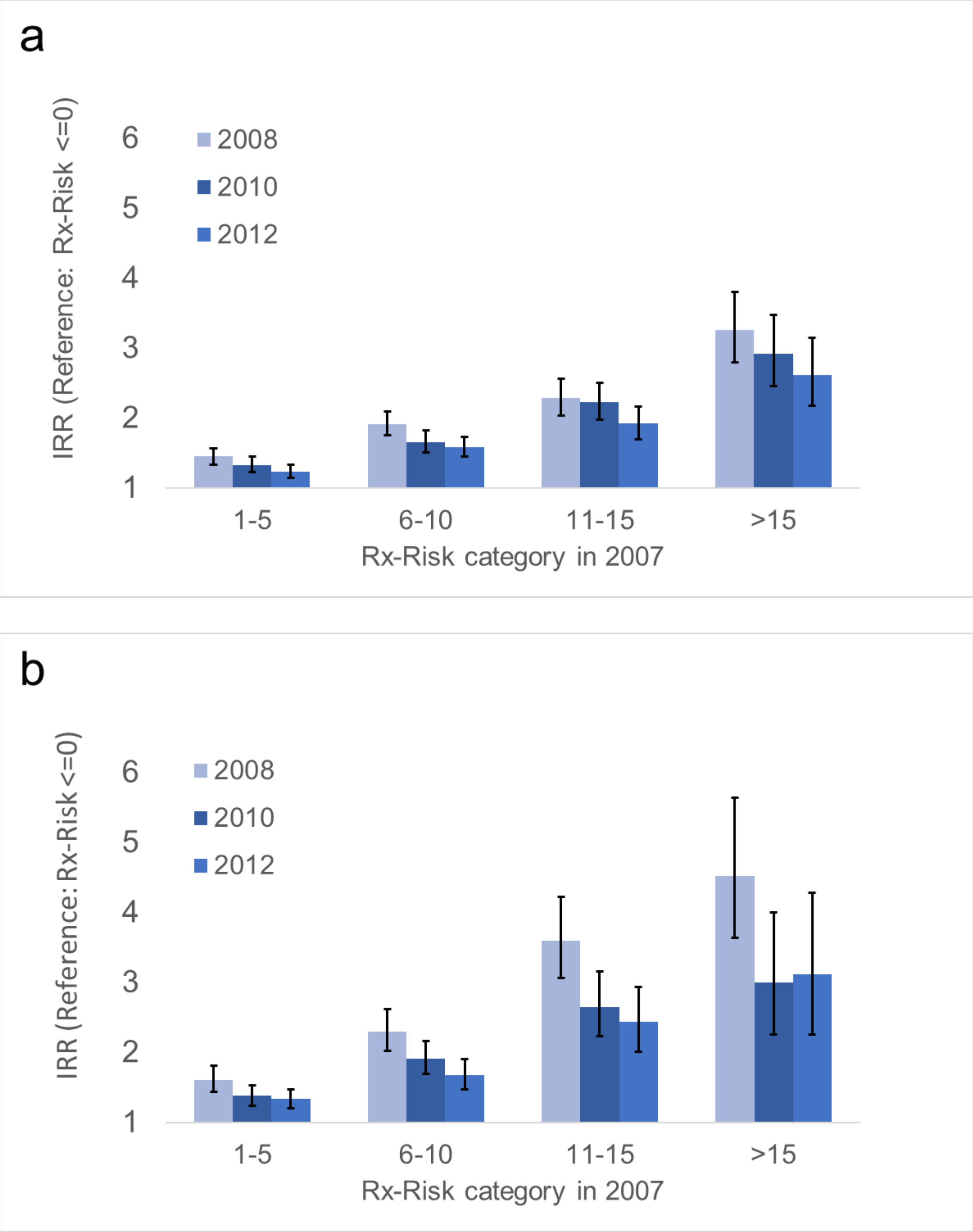

Supplementary Figure 3 Incidence rate ratio of hip fracture in women compared to men (reference) with 95% CI by Rx-Risk category and age group. Adjusted for birth year and calendar year. The Norwegian Prescription Database (2005-2016) and the NOREPOS Hip Fracture Database (2006-2017)

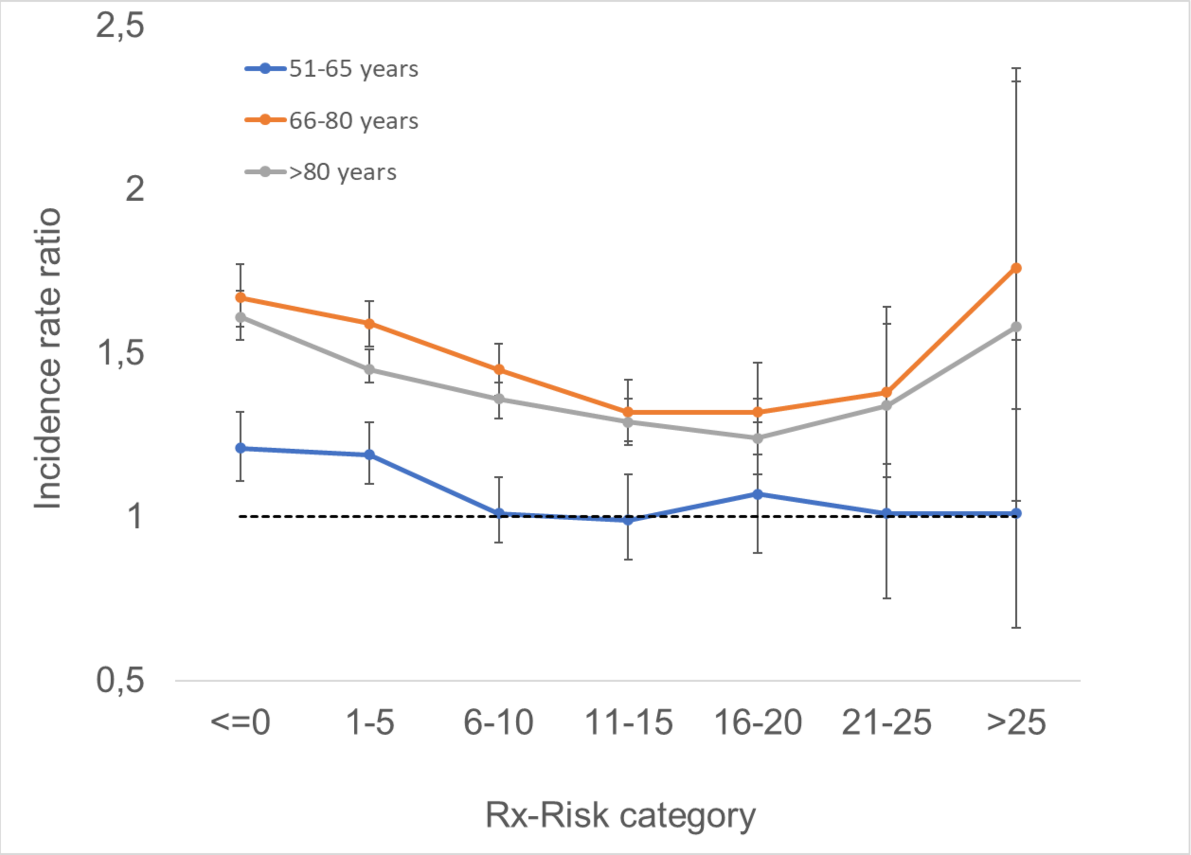

Supplement: Supplementary file 1 — Additional file 1: Supplementary Table 1-5. and Supplementary Figures 1-3. Supplementary Table 1. Disease categories included in the medication-based Rx-Risk Comorbidity Index and their assigned severity weights in 2011, based on individuals aged ≥ 50 years included in the Norwegian Prescription Database. Supplementary Table 2. Hip fracture incidence with 95% CIs by Rx-Risk category and age group in women and men. Supplementary Table 3. Risk of hip fracture in 2012 in women and men ≥ 51 years by disease categories in 2011 included in the Rx-Risk Comorbidity Index. Supplementary Table 4. Sensitivity analyses including individuals <70 years without an Rx-Risk in the reference group. Supplementary Table 5. Sensitivity analyses excluding individuals without an Rx-Risk. Supplementary Figure 1. Disease categories included in the medication-based Rx-Risk Comorbidity Index and their odds ratios for 1-year mortality and assigned severity weights during 2005-2016, based on individuals aged ≥ 50 years included in the Norwegian Prescription Database. Supplementary Figure 2. Latency analysis. Incidence rate ratios with 95% CIs for hip fracture in 2008, 2010 and 2012 by Rx-Risk category in 2007. Supplementary Figure 3. Incidence rate ratio of hip fracture in women compared to men by Rx-Risk category and age group. [file 12916_2024_3335_MOESM1_ESM.pdf]
